# Unrelated Helpers in a Primitively Eusocial Wasp: Is Helping Tailored Towards Direct Fitness?

**DOI:** 10.1371/journal.pone.0011997

**Published:** 2010-08-06

**Authors:** Ellouise Leadbeater, Jonathan M. Carruthers, Jonathan P. Green, Jasper van Heusden, Jeremy Field

**Affiliations:** 1 School of Life Sciences, University of Sussex, Brighton, United Kingdom; 2 Natural Environment Research Council (NERC) Biomolecular Analysis Facility, University of Sheffield, Sheffield, United Kingdom; University of Bristol, United Kingdom

## Abstract

The paper wasp *Polistes dominulus* is unique among the social insects in that nearly one-third of co-foundresses are completely unrelated to the dominant individual whose offspring they help to rear and yet reproductive skew is high. These unrelated subordinates stand to gain direct fitness through nest inheritance, raising the question of whether their behaviour is adaptively tailored towards maximizing inheritance prospects. Unusually, in this species, a wealth of theory and empirical data allows us to predict how unrelated subordinates should behave. Based on these predictions, here we compare helping in subordinates that are unrelated or related to the dominant wasp across an extensive range of field-based behavioural contexts. We find no differences in foraging effort, defense behaviour, aggression or inheritance rank between unrelated helpers and their related counterparts. Our study provides no evidence, across a number of behavioural scenarios, that the behaviour of unrelated subordinates is adaptively modified to promote direct fitness interests.

## Introduction

Nests of the primitively eusocial paper wasp *Polistes dominulus* are founded in the spring by one or a small group of overwintered reproductive females. In groups, one female becomes dominant and monopolizes reproduction, while subordinates forage to feed the brood [1], [2]. These co-foundress associations are unique amongst social insect breeding groups, because many subordinate wasps are completely unrelated to the dominant, but lay almost none of the eggs [3], [4], [5]. Given that foundresses survive for only one breeding season, breeding independently would seem to represent a better option than helping an unrelated wasp to breed, but the potential benefits of group membership become apparent when the possibility of nest inheritance is considered [6]. Foundresses live in small groups and queen mortality rates are high, so subordinates may have a significant chance of inheriting the dominant position [4], [7], [8], [9]. Thus, even though subordinates may obtain no *current* direct fitness while the dominant is alive, they have potential “future fitness” [9]. Whilst this might help to explain why unrelated subordinates join nests, it does not explain why they devote time and energy to brood care. Why should individuals forage for and protect brood in which they have no kin-selected interest, rather than simply waiting to inherit?

One possible explanation for helping by unrelated subordinates is that wasps cannot accurately discriminate kinship at the individual level. However, at least some *P. dominulus* co-foundresses must derive from different natal nests, because relatedness is typically lower in co-foundress associations than within broods on the previous season's nests from which they derive [4]. Inter-nest (*cf* intra-nest) kin discrimination is common in wasps [10], suggesting that at least some individuals should be recognized as non-kin. Furthermore, there is evidence that the chemical information necessary for discrimination even between different sister groups born on the same natal nest is present in this species [11]. Thus, it seems likely that unrelated subordinates may indeed recognize that they are not kin of the wasp whose offspring they help to rear.

In this paper, we test the hypothesis that the helping behaviour of non-relatives is tailored towards attaining future fitness. In other words, unrelated subordinates may choose to avoid participating in tasks that might compromise their chances of inheritance. The *P. dominulus* study system is unusual in that extensive previous work allows us to identify such contexts. For example, in many species, it might be difficult to predict whether foraging for unrelated brood will improve or worsen an individual's prospects of gaining future fitness through inheritance. On the one hand, rearing brood will increase group size, providing a larger workforce should inheritance occur, and possibly also boosting the helper's own chance of survival [12], [13], [14]. On the other, the energetic costs or mortality risks of helping might be substantial. In primitively eusocial wasps, however, prior work has found that the costs of foraging to future fitness most likely outweigh the benefits. In both *P. dominulus* [15] and *Liostenogaster flavolineata* [9], another small-group social wasp where inheritance is common, subordinates reduce their foraging effort when they attain higher ranks in the queue to inherit the nest. This supports theoretical findings [15], [16] that when future fitness is a realistic possibility, as it is for higher-ranked wasps, the high mortality risk of foraging leads to selection for reduced helping effort.

On this basis, we predict that since unrelated subordinates are under selection to maximize future fitness, they will forage less than their related counterparts. Queller and colleagues [4] have previously found a marginally significant tendency for more distant relatives of the dominant to spend less time foraging, in a small sample under laboratory conditions. Empirical data from *P. dominulus* also allow us to predict how unrelated subordinates should behave towards their nestmates. Cant and Field [17] found that wasps that were highly-ranked in the queue to inherit were more aggressive towards their nestmates, whilst Cant and colleagues [18] showed that Rank 2 wasps that stood to inherit larger groups were more likely to escalate experimentally-induced conflicts with the dominant. Both results are consistent with theoretical predictions that selection to maximize future fitness promotes aggression towards nestmates, especially the dominant wasp [18], [19], perhaps because aggressive behaviour might improve a wasp's position in the queue to inherit the nest [18].

In this study, we add two further behavioural contexts where we consider that helping might compromise future fitness. We investigate nest defense behaviour, because defending the nest from conspecific usurpers carries a risk of serious injury [20]. If unrelated subordinates can choose their own level of effort, they might be less willing to contribute to group nest defense. We also investigate whether unrelated subordinates occupy higher ranks in the queue to inherit the nest. In summary, we test the hypotheses that foraging effort, intra-nest aggression, nest defense behaviour, and inheritance rank, will vary according to a subordinate's relatedness to the dominant.

Parallels with the *P. dominulus* social system may be found amongst certain inheritance-based vertebrate co-operative groups, where attempts to link helping effort to relatedness have produced mixed results [21], [22], [23], [24]. Our invertebrate study system is unusual in that a wealth of theoretical and empirical work has demonstrated the effects of variation in future fitness on helping behaviour, allowing predictions about how unrelated subordinates should behave. In the following experiments, we make use of this opportunity to compare the helping behaviour of individuals to which kin-selected benefits can and cannot apply.

## Methods

### Behavioural studies

**Figure 1:**
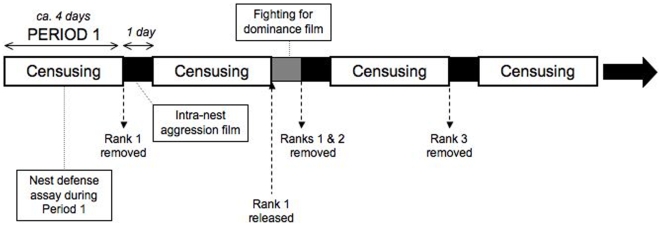
Sequence of experiments. Black/grey sections represent one day of no censusing. Observations continued until the rank of each wasp on the nest was known (pausing for bad weather).

In early March 2009, we selected 241 nests on hedges of *Opuntia* cactus running along the edges of a mixed arable/pasture site in Southern Spain. We individually marked (Humbrol paints) and clipped a tarsal sample (stored in 1 ml pure ethanol) from each foundress. For six weeks starting 1^st^ April, we selected groups of approximately six marked nests each day, each with 3–6 foundresses and large larvae or older brood. The sequence of experiments is summarized in Figure 1.

#### Work effort and inheritance rank

To ascertain whether unrelated subordinates forage relatively less, we visited each nest approximately every 45 minutes on sunny afternoons for four days (Period 1), recording which individuals were present (27±0.5 surveys per nest, mean ± standard error). Foraging effort was estimated as the proportion of surveys in Period 1 in which an individual was away from the nest.

Following [15], we identified the dominant (Rank 1) as the individual that was most often present on the nest. Where fewer than three surveys separated the closest contenders, we continued censusing each following day until this criterion was achieved. The next morning, Period 1 ended when we removed the Rank 1 wasp before 0800.

Censusing and successive removals continued until the ranks of all individuals had been ascertained (13–33 days), at which point the nest and remaining occupants were collected and frozen (−20°C). Inheritance rank estimates have a maximum error of one rank, because if one wasp died before its rank was known, we continued to estimate inheritance rank for the rest of the nest. If two wasps died, or if an individual died before the original dominant was identified, we did not estimate ranks any further for that nest. We obtained inheritance rank estimates for 177 subordinate wasps on 73 nests, and work effort data for 219 subordinates on 79 nests. 70 wasps died during the study period.

#### Nest defense

To assess whether unrelated subordinates participate in nest defense, we presented each nest with a dead conspecific “usurper” (from a distant site, killed by freezing) and filmed the reaction of the inhabitants, on sunny afternoons during Period 1. Usurpers were held with clean forceps, approximately 1 cm from the nest, for two minutes. We carried out assays on 75 nests.

Videos were scored using standard categories of aggression for this species [17]. We recorded “lunges” (leaping across nest, physical contact), “chews” (light biting), “grapples” (physical grasping) and “mounts” (climbing onto a nestmate). Subordinates' behaviour was classed as “aggressive” if they performed one or more of these acts towards the “usurper”.

#### Intra-nest aggression

To assess whether unrelated subordinates initiate/receive more aggression from nestmates, we filmed nests for four hours on the afternoon following early-morning removal of the Rank 1 (Figure 1). Rank 1 removal is a key moment for subordinates, because it represents an opportunity for inheritance. Interactions were scored as described above. For each wasp, we calculated the mean number of aggressive acts initiated and received per hour present during the film (n = 139 wasps on 49 nests).

#### Fighting for dominance

Unrelated subordinates may have a greater incentive to fight the dominant for control of the nest, but escalated fighting is rarely observed in undisturbed colonies. Following [17], we induced fights by returning the Rank 1 to the nest four days after her removal at the end of Period 1, filming the subsequent interaction between her and the new dominant (the Rank 2). Before they were returned, Rank 1 foundresses were stored in the refrigerator at 5°C and fed with 50% (v/v) sugar solution every two days. They were released approximately 1 m from the nest, on sunny afternoons only.

We filmed the return of 50 Rank 1 wasps, classing fights as “escalated” if they lasted for more than 4 seconds, and/or if they included a “falling fight” whereby both wasps fall from the nest whilst grappling [17]. The following morning, both the Rank 2 and Rank 1 were permanently removed so that the ranks of remaining wasps could continue to be ascertained (Figure 1).

### Relatedness estimation

#### Primer design

Microsatellite loci suitable for genotyping *P. dominulus* were identified by searching published literature and the EMBL sequence database. We used a selection of the primer sets previously isolated from *P. dominulus* and *P. bellicosus* [25], [26]. Inspection of the 28 *P. dominulus* microsatellite sequences described by Henshaw [26] revealed that 23 consisted of multiple cloned inserts (as they contained multiple GATC restriction sites). Details of which loci failed to amplify and the primer sets for these are not provided by Henshaw, but any primer sets designed to amplify across two inserts would be expected to fail. We therefore designed three new primer sets to amplify sequences that contained just a single microsatellite-containing insert and these were found to amplify successfully. Details of all 8 primers, including the new *P. dominulus* primer sequences are provided in Text S1.

For each of the 8 loci, we tested for Linkage Disequilibrium (Gamete Disequilibrium Test), for deviations from Hardy-Weinberg Equilibrium (exact HW test) and for heterozygote deficiency (expected if null alleles are present, U test) in a sample of 64 non-relatives from our study, using the software Genepop 4.0 [27]. We found no significant deviations from chance expectations in all cases (p> Bonferroni-adjusted sequential p-values based on 0.05).

#### DNA extraction and amplification

To extract genomic DNA, wasp tarsi were bathed in 50 µl of buffer solution containing 10 mM Tris-Cl (pH 8.2), 1 mM EDTA, 25 mM NaCl, and 200 µg/ml Proteinase K (adapted from [25]). Samples were incubated at 57°C for 40 minutes, then at 95°C for 2 minutes, to inactivate the Proteinase K.

Multiplex polymerase chain reactions were performed on a Peltier Thermal Cycler, using 8 fluorescently labeled microsatellite primer pairs (amplified in a single multiplex set). Reactions of 4 µl were performed, containing approximately 80 ng of the template DNA, 0.75 µmol of three primer pairs (Pdom1jc, Pdom2jc and Pdom20), 0.375 µmol of the remaining five primer pairs (Pdom7, Pdom140, Pbe128TAG, Pdom127b and Pdom25), and 2 µl PEQlab hot start mix Y (1.25 u “Hot” Taq DNA polymerase per 25 µl, 0.4 nM dNTPs, 40 mM Tris-HCl, 32 mM (NH_4_)_2_SO_4_, 0.02% Tween 20 and 4 mM MgCl_2_). The temperature profile for the amplification was 95°C for 15 minutes; 35 cycles of 94°C for 30 seconds, 57°C for 90 seconds and 72°C for 60 seconds; followed by a final extension step of 60°C for 30 minutes. A drop of mineral oil was added to prevent evaporation. Each plate included a positive and negative control to check for consistency of amplification.

PCR products were separated by size using a 48-well capillary Applied Biosystems 3730 Sequencer, compared with a size standard (Applied Biosystems GeneScan LIZ 500) and visualized using Applied Biosystems GeneMapper analysis software. We re-ran a subsample of 25 wasps to check for consistency of amplification and genotyping, and found that our genotyping error rate was low (only 1 incongruency between runs, in which a heterozygotic locus appeared homozygotic in one run).

#### Relationship assignment

We used the Full Sibship Reconstruction procedure in the program Kingroup (www.kingroup.org [28]) to establish each subordinate's relationship to the dominant. This procedure can divide groups of co-foundresses found on a nest into discrete sub-groups of full siblings- “sister groups”- based on the likelihood that all pairs of individuals within each sister group are sisters, and all pairs in different sister groups are related at the level of cousins or less. For example, consider a group of four co-foundresses that comprised two sisters, one cousin of these sisters and one unrelated wasp. The program would place the two sisters within the same sister group, and the cousin and unrelated wasps in two separate groups, producing three groups in total. Kingroup's allocation of pairs to “sister” or “cousin” categories is based on the likelihood that the genotypes of the two individuals would occur if they were full sisters, versus the likelihood that they would occur if the individuals were maternal cousins, given the population allele frequencies [29]. We provide a description of the iterative steps of the Full Sibship Reconstruction procedure that Kingroup uses in Text S2.

One we had divided cofoundresses from each nest into sister groups, we used the same procedure to identify cousin groups. In this case, the program follows exactly the same steps, but finds the pairwise likelihoods that individuals are cousins *vs*. unrelated. Thus, to summarize, for each nest, we knew which wasps were likely to be sisters of one another, which were likely to be cousins, and which were unrelated. Since we knew the dominant's identity, we could hence classify all subordinate wasps as sisters, cousins or non-relatives of the dominant wasp, and these three categories were used as predictors of behaviour in all subsequent analyses. However, since cousins and sisters most likely derive from the same natal nest, we also re-ran the same analyses where “sisters” and “cousins” were grouped as one category. Our findings did not change.

### Statistical analyses

#### Behavioural data

Behavioural data were analyzed using the software R (2008 [30]). Our findings can be divided into two types of analyses: those where the data include more than one individual from each nest (individual foraging effort, responses to potential usurpers, and intra-nestmate aggression received/initiated), and those where each nest contributes only one value to the dataset (total nest foraging effort, total intra-nestmate aggression, occurrence of escalated fighting on return of Rank 1 wasp).

In the former case, data from individuals on the same nest cannot be considered independent, so we used mixed models where “nest” could be fitted as a random factor, to avoid pseudoreplication. For data where the error distribution of the variable being tested was expected to be binary (e.g. aggressive response *vs.* no response), we fitted generalized linear mixed models that assume a binomial error structure (“lmer”); otherwise, we fitted linear mixed effects models (“lme,” suitable for a normal error distribution).

For those analyses that did not involve data from multiple wasps on the same nest, we used linear models. Again, where the response variable was binary, we used a model type suitable for a binomial error structure (generalized linear model, “glm”); otherwise, we used linear models (“lm”). Proportional data (foraging effort) were arcsine transformed prior to analysis. A full list of the fixed effects included in each model, and a description of the model type for each behaviour tested, can be found in Text S3.

In each case, we began by fitting the full model, and proceeded by dropping the least significant terms sequentially until further removal led to a significant (p<0.05) decrease in the explanatory power of the model. This was assessed by comparing the models with and without the term in question, using Log-likelihood tests for linear mixed models (here the test statistic is a likelihood ratio *L*, which closely approximates a χ^2^ distribution with *v* degrees of freedom, where *v* is the difference in the number of parameters between the two models), χ^2^ values for models with a binomial error structure, and tabulated values of F-values for linear models with normal errors. To establish the final significance levels for each term, we added (non-significant) or removed (significant) terms to/from the minimal model. Non-significant terms (p>0.05) are not reported unless relevant to the main hypotheses.

We did not include inheritance rank as a predictor of behaviour in our analyses, because the rank of wasps on the same nest cannot be considered independent, since no position can be occupied by more than one wasp. However, since inheritance rank influences foraging effort and aggression [16], [31], [32], we first established that unrelated wasps did not occupy consistently different ranks to other subordinates (regression of relatedness to the Dominant against rank; Spearman's ρ). Inclusion of rank as a predictor of behaviour in the analyses did not alter the results. Data from foundresses of all ranks were included in the analyses.

#### Population relatedness

We carried out Maximum Likelihood analysis to ascertain the population composition, in terms of sister, cousin and unrelated pairs, that most closely matched the distribution of relatedness in our sample. Kingroup can produce distributions of pairwise relatedness values for simulated populations containing only individuals of a specified relatedness (e.g. sisters) based on user-defined population allele frequencies [28]. We created separate pools of haplodiploid sisters, cousins and non-relatives (n>4000 in each pool) based on our observed population allele frequencies, and then pseudo-randomly sampled from them to create relatedness distributions for populations of known composition. For example, to create the relatedness distribution of a population containing 75% sisters, 20% cousins and 5% non-relatives, we sampled pairwise relatedness values from the three pools in those proportions. We compared the 232 relatedness distributions created in this way (the composition of sisters, cousins, and non-relatives varying at 5% intervals between distributions) to our observed distribution using Kolmogorov-Smirnov tests. The population where the match was closest was identified by the highest p-value.

## Results

### Relatedness estimation

**Figure 2:**
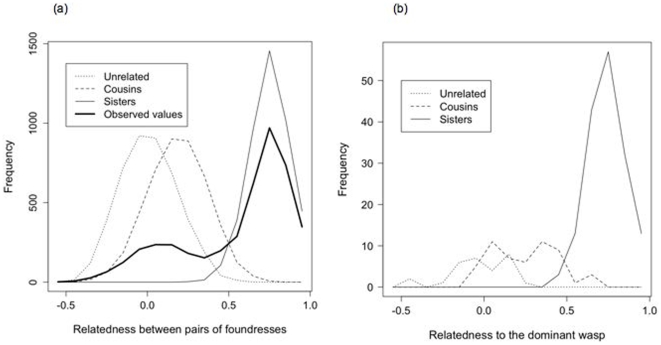
Relatedness in the study population and sample. a) Distribution of pairwise nest-mate relatedness across whole population, based on a sample of 4396 cofoundress pair (broad line). Further lines represent pairwise relatedness from simulated populations comprising 4396 pairs of sisters, cousins, and unrelated wasps b) Distribution of relatedness to the dominant wasp for subordinates classed as sisters of the dominant, cousins of the dominant, and non-relatives, on nests used for behavioural observations. Categories overlap because allocations are based on pairwise likelihoods, which depend on the population allele frequencies, and not absolute cut-off values. For example, an individual that is related to the dominant by less than 0.1 might be found to be more likely a cousin than unrelated, if the particular alleles that the two individuals do share are rare in the population.

The distribution of within-nest, pair-wise relatedness across our entire DNA-sampled population (241 nests) showed a large peak around the full haplodiploid sister value of 0.75, and a smaller, broad peak centered at approximately 0.1 (Figure 2a). This distribution is similar to that found previously in the Italian population studied by Queller et al. [4], although the proportion of non-sibling pairs is lower in our population. Maximum likelihood analysis reveals that our population most likely contains at least 15% unrelated pairs, with the remainder comprising 15% cousins and 70% full-sibling pairs. This population structure was more likely that any population containing 5% or fewer unrelated pairs by a factor of 1×10^3^ and any population containing no unrelated pairs by a factor of more than 7×10^3^. Thus, like the Italian population, our Spanish population contains a significant proportion of unrelated co-foundresses.

For the 72 nests used in behavioural observations, 12% of subordinates were classed as unrelated to the dominant, 66% as sisters of the dominant, and 22% as cousins (Figure 2b). 24.7% of nests contained at least one subordinate that was unrelated to the dominant wasp, and mean within-nest relatedness was 0.54±0.015 (mean ± standard error). Nests containing unrelated foundresses did not differ significantly in number of co-foundresses from those containing only one sister group (*t_76_* = 0.09, *p* = 0.92). Wing length (as a proxy for body size) did not differ significantly between sisters, cousins, and non-relatives of the dominant (means ± standard error: sisters: 11.7 mm ±0.06, cousins: 11.6±0.09, unrelated: 11.72±0.13, lme: L_2_ = 0.36 *p* = 0.91).

### Behavioural data

#### Inheritance rank

**Figure 3:**
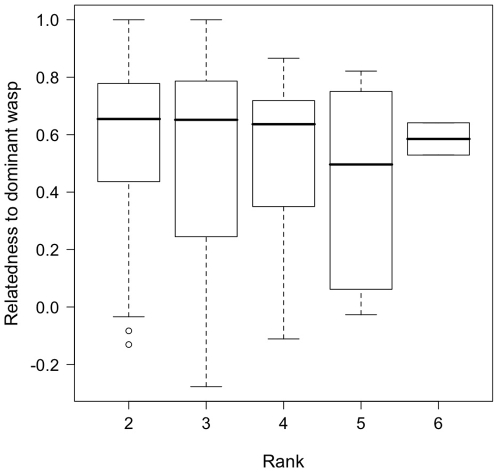
Inheritance rank in relation to a subordinate's relatedness to the dominant wasp. Medians, interquartile range and max/min values are indicated.

We found no significant correlation between subordinates' inheritance ranks and relatedness to the dominant wasp on the nest (Figure 3, Spearman's ρ = −0.11, p = 0.13). The dominant position was occupied by wasps with no relatives in the group no more often than would be expected by chance (χ^2^ (Yates' correction)  = 0.02, d.f. = 1, p>0.01).

#### Work effort

The total work effort on nests containing unrelated subordinates did not differ from nests that contained only sisters and cousins of the dominant (lm, F_1,75_ = 0.03, p>0.85). Only the date (work levels dropped later in the season) and, as expected, group size (larger nests had higher total work effort) significantly influenced total work effort on a nest (F_1,76_ = 9.91 and F_1,76_ = 99.32 respectively, p<0.01 in both cases).

**Figure 4:**
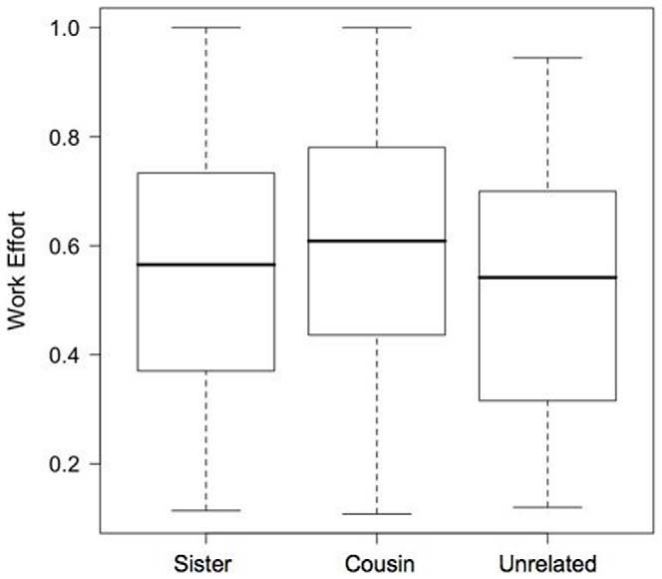
Foraging effort of sisters, cousins and non-relatives of the dominant wasp. Foraging effort is estimated based on proportion of time spent away from the nest.

For individual wasps, a subordinate's relationship to the dominant wasp did not significantly influence work effort (Figure 4, lme: L_1_ = 0.003 p>0.95). Again, wasps worked harder earlier in the season (lme: L_1_ = 6.87, p<0.1), and when in smaller groups (lme: L_1_ = 4.02, p<0.05). Including inheritance rank in the analysis (but see Methods) did not change these results.

We observed instances where aggression from a nestmate immediately preceded departure on 17 nests, but this was no more likely to occur on nests containing unrelated subordinates than other nests (χ^2^ (Yates' correction)  = −1.75, d.f. = 1, p>0.9).

#### Nest defense

**Figure 5:**
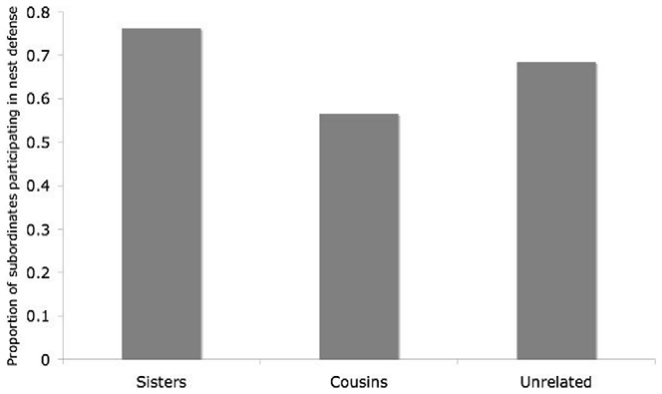
Aggressive responses to a conspecific usurper by sisters, cousins and non-relatives of the dominant.

80% of wasps that were present during the assay defended their nest. We found no significant differences between unrelated subordinates, cousins, and sisters of the dominant (lmer: χ^2^ = 2.80, d.f. = 1, p = 0.08). Of the three groups, cousins of the dominant, rather than unrelated wasps, responded the least aggressively (Figure 5). Smaller wasps were more likely to respond aggressively (lmer, χ^2^ = 13.74, d.f. = 1, p<0.01).

#### Intra-nest aggression

**Figure 6:**
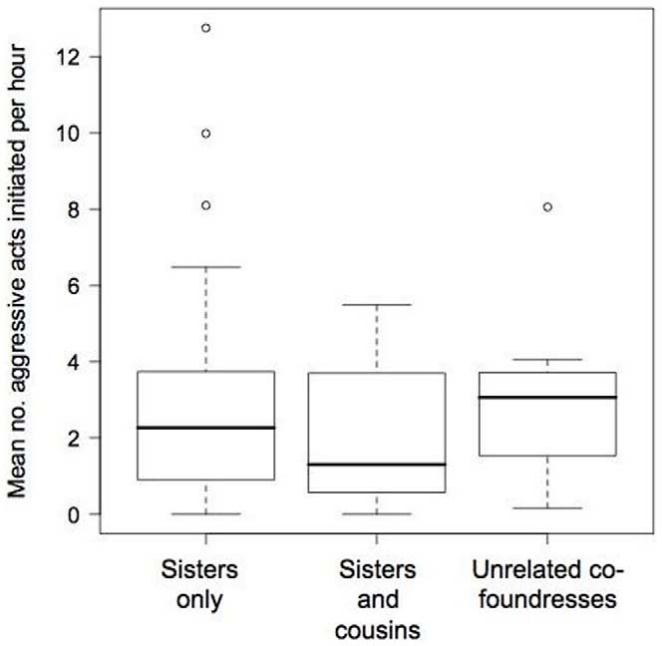
Aggression levels within founding groups. Mean aggression rates on nests where co-foundresses were all sisters, cousins and sisters, or contained at least one wasp that was not related to the rest of the group are shown.

When the dominant was removed, the subsequent level of aggression (mean aggressive acts initiated per wasp, per unit time) as wasps re-established the social hierarchy was no higher on those nests that contained unrelated subordinates than other nests (lm: F_1,48_ = 0.01, p = 0.94, Figure 6). Wasps with no sisters in the group (i.e. unrelated group members) neither received (lme: L_1_<0.00, p = 0.99) nor initiated (lme: L_1_ = 3.09, p = 0.08) significantly more aggressive acts than other wasps. Aggression that led recipients to temporarily leave the nest was rare (17 instances in over 1200 hours of video footage) and was no more likely to occur on nests containing unrelated subordinates than other nests (χ^2^ (Yates' correction)  = −1.75, d.f. = 1, p>0.9).

#### Fighting for dominance

When fights between Rank 1 and Rank 2 wasps were experimentally induced, unrelated Rank 2 subordinates escalated conflict no more often than other wasps (glm: χ^2^ = 2.08, d.f. = 1,p = 0.15). When escalated fighting took place, the returning Rank 1 won the fight in all but a single case, on a nest where the Rank 1 and Rank 2 were sisters.

## Discussion

Unrelated subordinates behaved like other members of *P. dominulus* social groups, and their presence did not affect group function across a wide range of contexts, in a natural habitat, with large sample sizes. Thus, we found no evidence that helping investment reflected a subordinate's relationship to the dominant wasp.

Our data provide no support for the hypothesis that we set out to test- that the helping behaviour of unrelated subordinates is tailored towards maximizing future fitness in *P. dominulus*. Given that reproduction through inheritance represents the only source of fitness for unrelated subordinates, what other hypotheses might explain why they raise unrelated brood? We discuss four alternatives.

First, selection to maximize direct fitness might have similar outcomes to selection to maximize indirect fitness. In other words, the same behaviours that boost the fitness of the current brood (and thus indirect fitness, for relatives of the dominant) may also boost future fitness. To re-visit an example discussed in the introduction, foraging to feed the dominant's brood might boost future fitness, by increasing group size and thus the helper's survival prospects. However, in our study, we specifically included behavioural contexts for which there is evidence that the fitness interests of unrelated and related subordinates are not aligned. We know from previous work that higher ranked wasps, that have greater expected future fitness, forage less [9], [15], behave more aggressively towards nestmates [17], and challenge the dominant for control of the nest [18]. This provides a strong basis to suggest that the same trends should be apparent in the behaviour of unrelated subordinates, which are also under selection to maximize direct fitness.

A related possibility is that relatives of the dominant may stand to gain little indirect fitness through raising spring brood, which contains a high proportion of non-reproductive workers [1]. Thus, both related and unrelated subordinates might be under selection to maximize future fitness, and we might see little difference in their behaviour. However, a substantial proportion of spring brood do indeed reproduce, because on some nests all foundresses die (approximately 23%, Leadbeater and Field unpublished data) and a worker can thus attain the dominant position [1]. In addition, many spring brood are male offspring, and thus reproductive [1]. Relatives of the dominant hence stand to gain indirect fitness through helping even on spring nests, albeit relatively less than on summer nests.

A third alternative is that unrelated helpers are not free to choose their own level of help, but must “pay” for group membership [33]. The dominant may be selected to evict unrelated helpers who might otherwise inherit in place of a relative, unless their elevated work effort justifies their presence. Can dominants evict subordinates, or otherwise enforce helping, in *P. dominulus*? Aggression that immediately preceded a subordinate leaving the nest to forage was rare in our study, and was equally directed towards relatives and non-relatives. However, perhaps actual evictions are not observed because the threat of eviction effectively motivates helping behaviour. Put differently, perhaps unrelated subordinates *would* be evicted if they did not work hard enough, but because this threat is effective, they do work hard and we do not see evictions [34]. Nonetheless, if this were the case, we should expect unrelated subordinates to work harder than relatives of the dominant, because the cost of their presence (a place in the inheritance queue that could otherwise have been occupied by a related subordinate) is higher. Thus, while our findings do not support the hypothesis that subordinates freely choose to maximize future fitness by working *less* hard, nor are they consistent with the hypothesis that unrelated subordinates pay-to-stay.

A final alternative is that helpers may make kin recognition errors. If unrelated co-foundresses derive from the same natal nest as their co-foundresses, kin recognition may be challenging. As we highlight in the introduction, although intranidal kin discrimination is rare in social insects, internidal discrimination is common [10], and at least some unrelated subordinates must derive from different natal nests to their co-foundresses [4]. Why should individuals not be capable of recognizing these outsiders as non-relatives? A possibility is that the hydrocarbon profiles of wasps overwintering together may become indistinguishable by the spring [35], since winter refuges are sometimes shared with individuals from other nests [36]. Relatedness in spring nests is not lower than within hibernaculae [5], suggesting that unrelated foundresses could plausibly be hibernaculum-mates of their co-foundresses, but this raises the question of why other *Polistes* do not share this problem [37]. Nonetheless, kin recognition errors between hibernaculum-mates provide a plausible explanation for our findings, and further investigation of the source of unrelated foundresses is already underway.

## Supporting Information

Text S1 Primer sequences. Details of primers used in this study, including three new primer sets.
(0.05 MB DOC)

Text S2 Full Sibship Reconstruction procedure. Description of the iterative procedure followed by Kingroup during Full Sibship Reconstruction.
(0.29 MB DOC)

Text S3 Statistical models. Descriptions of fixed effects and model types for each statistical model.
(0.10 MB DOC)
