# Supplementary material for: Unrelated Helpers in a Primitively Eusocial Wasp: Is Helping Tailored Towards Direct Fitness?
Source: PLoS One. 2010 Aug 6;5(8):e11997. doi: 10.1371/journal.pone.0011997 (PMC2917371; doi:10.1371/journal.pone.0011997)
Supplement: Text S1 — Primer sequences. Details of primers used in this study, including three new primer sets. (0.05 MB DOC) [file pone.0011997.s001.doc]

*S1: Characterisation of eight microsatellite loci in Polistes dominulus, including details of three new primer sets.* New primer sets were designed for three *Pdom* loci because the original primer sets failed to amplify any product.

| **Locus** | **EMBL accession number** | **Ref.** | **Repeat motif observed in source species** | **Primer sequences (5’-3’) and names of the new primer sets developed** | **N^1^** | **Expected allele size (bp)** | **Observed allele sizes (bp)** | **No. of alleles** | ***H*_O_** | ***H*_E_** | **Dye^2^** |
| --- | --- | --- | --- | --- | --- | --- | --- | --- | --- | --- | --- |
| Pbe128TAG | U64637 | A | (TAG)_12_ | F: CCGATATCCGTGCCAGTGATAC  R: GCTACCGCGACTGCTGTCC | 26 | 170 | 128-197 | 15 | 0.81 | 0.83 | NED |
| Pdom1 | AF155596 | B | Pdom1jc (new primer set) (CAG)_9_TAG(CAG)_5_  (CAT)_5_GGCAC(CAG)_3_ | F: TCGGCTGATTTGTCAATACG  R: ATTTAATCGCGAACGGTGTC | 26 | 275 | 243-290 | 19 | 0.69 | 0.76 | 6-FAM |
| Pdom2 | AF155597 | B | Pdom2jc (new primer set) (AAG)_8_CG(AAG)_2_ | F: AGACCCACCAGCTCCTCTC  R: TCTTCGTTCCTTAAGCTTACAATG | 26 | 180 | 165-191 | 11 | 0.58 | 0.69 | 6-FAM |
| Pdom7 | AF155598 | B | (CAG)_9_TAG(CAG)_5_  (CAT)_5_GGCAC(CAG)_3_ | F: CACTGTATTGTCCTACGGTGGTCC  R: GCGAGAACCTGTACTCAAAACAAAC | 23 | 160 | 154-179 | 9 | 0.65 | 0.67 | PET® |
| Pdom20 | AF155599 | B | (CAT)_18_ | F: TTCTCTGGCGAGCTGCACTC  R: AGATGGCATCGTTTGAAAGAGC | 27 | 236 | 210-372 | 29 | 0.89 | 0.91 | NED |
| Pdom25 | AF155600 | B | Pdom25jc (new primer set) (AAG)_11_ | F: CCGCGTTAACGATGAATG  R: TGGAAACGTAAGTCCACTCG | 26 | 142 | 120-147 | 9 | 0.69 | 0.65 | 6-FAM |
| Pdom127b | AF155610 | B | (AAT)_13_...(AAT)_6_ AA  (AAT)_4_AAC(AAT) | F: TCCCCCGTTTTTGGTCCTTG  R: GGGAGAGAATCGTGCCTTTTC | 24 | 119 | 106-171 | 18 | 0.92 | 0.90 | VIC |
| Pdom140 | AF155613 | B | (TAG)_9_ | F: GCTTTTCCCTTATTTTCCCG  R: CGTGTTCGTATATTCCTGTAACG | 27 | 192 | 196-244 | 21 | 0.85 | 0.84 | PET® |

^1^Number of *Polistes dominulus* individuals genotyped for primer development, belonging to a single population collected at Conil de la Frontera, Cadiz, Spain.

^2^Applied Biosystems Standard Dye Sets

**References** for the isolation of the original microsatellite sequence and primer set:

A: Strassmann, J. E., Barefield, K., Solis, C. R., Hughes, C. R. & Queller, D. C. 1997 Trinucleotide microsatellite loci for a social wasp, Polistes. *Molecular Ecology* **6**, 97-100.

B: Henshaw, M. T. 2000 Microsatellite loci for the social wasp *Polistes dominulus* and their application in other polistine wasps. *Molecular Ecology* **9**, 2155-2157.
