# Supplementary material for: Unrelated Helpers in a Primitively Eusocial Wasp: Is Helping Tailored Towards Direct Fitness?
Source: PLoS One. 2010 Aug 6;5(8):e11997. doi: 10.1371/journal.pone.0011997 (PMC2917371; doi:10.1371/journal.pone.0011997)

*S2: Full Sibship Reconstruction procedure- illustration*

For each nest, this iterative procedure starts with only one of the individuals in the nest group (Wasp 1), and moves forward by introducing the next focal wasp (Wasp 2). Two alternative sister groupings then become possible- one in which both wasps are in the same sister group, and another in which they are in separate groups. The program assesses which grouping is more likely by calculating the likelihood that the two wasps are sisters, based on their genotypes and the population allele frequencies, compared with the likelihood that they are related at the level of cousins or less. The most likely grouping (i.e. Wasp 1 and Wasp 2 in separate sister groups, or in the same group) is then carried forward to the next iteration, whereby the next focal nestmate is introduced (Wasp 3). The program finds all possible new groupings that could be created by introducing Wasp 3 to the sister groups carried forward from the previous iteration, or by placing Wasp 3 in a sister group on its own. Again, it calculates the likelihood of each grouping, based on the product of the likelihoods that pairs of wasps in the same allocated sister group are sisters, and are unrelated to those in different allocated sister groups. This is summarized in Figure S1. The iterations terminate when all the nestmates have been introduced.

Figure S1:


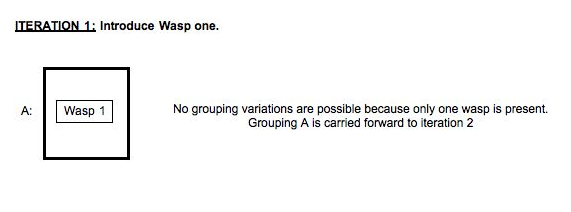


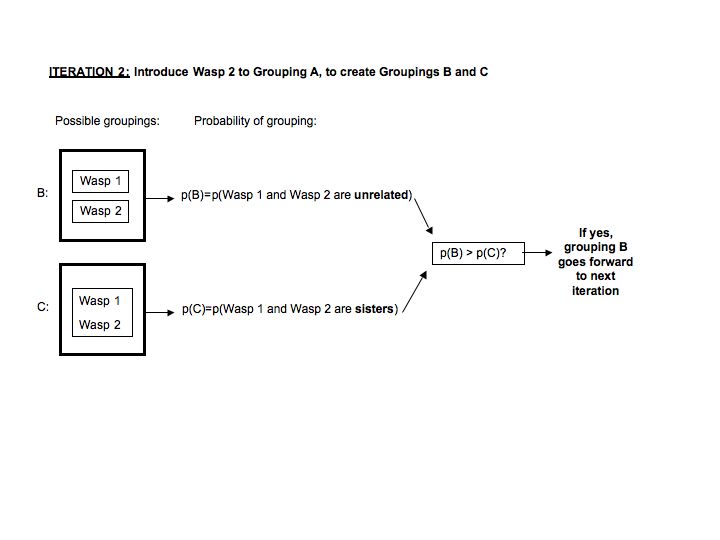


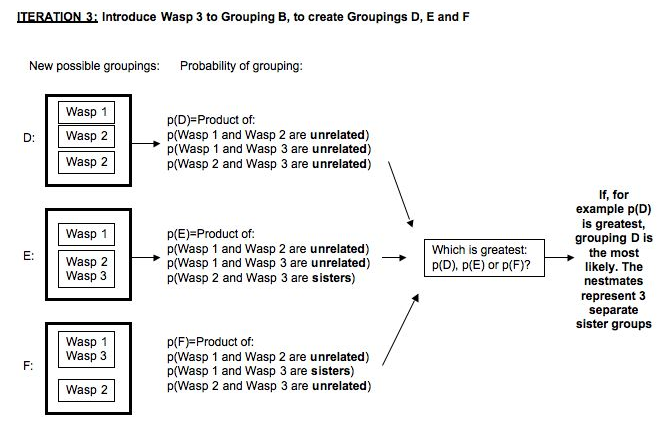

Supplement: Text S2 — Full Sibship Reconstruction procedure. Description of the iterative procedure followed by Kingroup during Full Sibship Reconstruction. (0.29 MB DOC) [file pone.0011997.s002.doc]
