# Supplementary material for: Unrelated Helpers in a Primitively Eusocial Wasp: Is Helping Tailored Towards Direct Fitness?
Source: PLoS One. 2010 Aug 6;5(8):e11997. doi: 10.1371/journal.pone.0011997 (PMC2917371; doi:10.1371/journal.pone.0011997)
Supplement: Text S3 — Statistical models. Descriptions of fixed effects and model types for each statistical model. (0.10 MB DOC) [file pone.0011997.s003.doc]

*S3: Summary of significance levels for fixed effects*

| **Dependent variable** | **Model type** | **Independent variable** | **Test statistic** | **p** | **Significance level**   - **<0.05** - ****<0.01** - *****<0.001** |
| --- | --- | --- | --- | --- | --- |
| Total nest foraging effort (sum of individual foraging efforts) | Linear model | Date | F | 0.002 | ** |
|  |  | Group size | F | 0.000 | *** |
|  |  | Nest type | F | 0.853 | ns |
|  |  | Mean foundress body size | F | 0.067 | ns |
| Individual foraging effort (proportion of surveys away from nest) | Linear mixed effects model | Date | Log-likelihood | 0.009 | ** |
|  |  | Group size | Log-likelihood | 0.045 | * |
|  |  | Body size | Log-likelihood | 0.700 | ns |
|  |  | Relatedness status | Log-likelihood | 0.954 | ns |
| Aggressive response to potential usurper (yes/no) | Generalized linear mixed effects model | Date | Log-likelihood | 0.197 | ns |
|  |  | Group size | Log-likelihood | 0.780 | ns |
|  |  | Body size | Log-likelihood | 0.000 | *** |
|  |  | No. of wasps present | Log-likelihood | 0.693 | ns |
|  |  | Relatedness to dominant | Log-likelihood | 0.094 | ns |
| Number of aggressive interactions per hour | Linear model | Date | F | 0.144 | ns |
|  |  | Group Size | F | 0.959 | ns |
|  |  | Nest type | F | 0.938 | ns |
|  |  |  |  |  | ns |
| Aggressive acts received per wasp | Linear mixed effects model | Date | Log-likelihood | 0.189 | ns |
|  |  | Group Size | Log-likelihood | 0.963 | ns |
|  |  | Relatedness | Log-likelihood | 0.079 | ns |
|  |  | Body Size | Log-likelihood | 0.066 | ns |
| Aggressive acts initiated per wasp | Linear mixed effects model | Date | Log-likelihood | 0.366 | ns |
|  |  | Group Size | Log-likelihood | 0.268 | ns |
|  |  | Relatedness | Log-likelihood | 0.986 | ns |
|  |  | Body Size | Log-likelihood | 0.349 | ns |
| Occurrence of escalated fighting on return of Rank 1 wasp | Generalized linear model | Relatedness | χ^2^ | 0.149 | ns |
|  |  | Group size | χ^2^ | 0.263 | ns |
|  |  | Relative size difference | χ^2^ | 0.596 | ns |
|  |  | Date | χ^2^ | 0.629 | ns |
